# An EGFR inhibitor enhances the efficacy of SN38, an active metabolite of irinotecan, in SN38-refractory gastric carcinoma cells

**DOI:** 10.1038/bjc.2011.397

**Published:** 2011-10-13

**Authors:** M Yashiro, H Qiu, T Hasegawa, X Zhang, T Matsuzaki, K Hirakawa

**Affiliations:** 1 Department of Surgical Oncology, Osaka City University Graduate School of Medicine, 1-4-3 Asahi-machi, Abeno-ku, Osaka 545-8585, Japan; 2 Oncology Institute of Geriatrics and Medical Science, Osaka City University Graduate School of Medicine, 1-4-3 Asahi-machi, Abeno-ku, Osaka 545-8585, Japan; 3 Oncology Center of Tongji Hospital, Tongji Medical College, Huazhong University of Science and Technology, Wuhan, PRC; 4 Department of Medical Oncology, Beijing Cancer Hospital, School of Oncology, Peking University, Beijing, PRC

**Keywords:** gastric cancer, chemoresistance, irinotecan, EGFR inhibitor, combination therapy

## Abstract

**Background:**

Acquired drug resistance to irinotecan is one of the significant obstacles in the treatment of advanced gastric cancer. This study was performed to clarify the effect of epidermal growth factor receptor (EGFR) inhibitors in combination with SN38, an active metabolite of irinotecan, on the proliferation of irinotecan-refractory gastric cancer.

**Methods:**

Two irinotecan-resistant gastric cancer cell lines, OCUM-2M/SN38 and OCUM-8/SN38 were, respectively, established by stepwise exposure to SN38 from the parent gastric cancer cell lines OCUM-2M and OCUM-8. The combination effects of two EGFR inhibitors, gefitinib and lapatinib, with SN38 on proliferation, apoptosis, and cell cycle on gastric cancer cells were examined.

**Results:**

Gefitinib or lapatinib showed synergistic anti-tumour effects against OCUM-2M/SN38 and OCUM-8/SN38 cells when used in combination with SN38, but not against OCUM-2M or OCUM-8 cells. SN38 increased the expression of EGFR and HER2 in OCUM-2M/SN38 and OCUM-8/SN38 cells. The combination of an EGFR inhibitor and SN38 significantly increased the levels of apoptosis-related molecules, caspase-6, p53, and DAPK-2, and resulted in the induction of apoptosis of irinotecan-resistant cells. The EGFR inhibitors increased the S-phase and decreased the UGT1A1 and ABCG expression in irinotecan-resistant cells. The SN38 plus Lapatinib group more effectively suppressed *in vivo* tumour growth by OCUM-2M/SN38 cells than either alone group.

**Conclusion:**

The combination treatment with an EGFR inhibitor and irinotecan might produce synergistic anti-tumour effects for irinotecan-refractory gastric cancer cells. The regulation of SN38 metabolism-related genes and cell cycle by EGFR inhibitors might be responsible for the synergism.

Gastric cancer remains a major health threat worldwide, and most patients with the advanced-stage disease require chemotherapy. Camptothecin (CPT), a DNA topoisomerase I inhibitor, blocks the DNA religation of topoisomerase cleavage complexes (Pommier *et al*, 1998), and is one of the effective anti-cancer drugs used in the chemotherapy of many types of solid tumours (Farhat, 2007; Segal and Saltz, 2009). Irinotecan hydrochloride (CPT-11) is currently clinically used as one of the preferred choices in monotherapy or combination therapy in advanced gastric cancer (Bugat, 2003; Arnold *et al*, 2006; Farhat, 2007). Also, irinotecan-based chemotherapy is recommended as a second-line treatment for gastric cancer patients according to the guideline of the National Comprehensive Cancer Network (1998). Although the development of drug resistance to CPT-11 is one of the greatest obstacles, a few effective therapies to combat chemoresistance to CPT-11 are currently available (Xu and Villalona-Calero, 2002).

With respect to advanced gastric cancer, a combination therapy using a molecular targeting compound and a chemotherapeutic inhibitor might achieve a better response rate not only for chemonative carcinomas but also for chemorefractory carcinomas, compared to therapy with a chemotherapeutic inhibitor alone. However, the effects of such a combination in patients with advanced gastric cancer remain to be clarified.

The human epidermal receptor (HER) family consists of four closely related transmembrane receptors: the HER1/epidermal growth factor receptor (EGFR), HER2, HER3, and HER4. Epidermal growth factor signals participate in the regulation of a variety of cell functions, including cell differentiation, proliferation, apoptosis, migration, and angiogenesis (Kishida *et al*, 2005; Kim *et al*, 2008). Epidermal growth factor receptor is highly expressed in approximately one-third of advanced-stage gastric cancers (Nicholson *et al*, 2001; Rojo *et al*, 2006; Galizia *et al*, 2007; Liakakos *et al*, 2008), and the activation of EFGR may be involved in chemoresistance in solid tumours (Ciardiello and Tortora, 2001). Recently, small molecules acting as tyrosine kinase inhibitors (TKIs) have begun to emerge as potential therapeutics for use in combination with cytostatic drugs in various types of carcinomas. Two studies have reported that an EGFR inhibitor in combination with cytostatic drugs achieved a better response rate for chemorefractory colorectal carcinomas than the cytostatic drugs alone (Cunningham *et al*, 2004; Saltz *et al*, 2004). Although preliminary data indicate that gefitinib monotherapy has limited efficacy for the treatment of gastric carcinoma (Yokoyama *et al*, 2006), preclinical data have shown the benefit of TKIs, including gefitinib, especially in combination with conventional cytostatic therapy (Becker *et al*, 2006a). A phase II study showed that gefitinib alone did not show clinical benefit in gastric carcinomas (Knight *et al*, 2004), while there was a significant correlation between increased exposure to gefitinib and enhanced apoptosis (Rojo *et al*, 2006). In certain gastric cancer cell lines, SN38, an active metabolite of irinotecan, has been shown to induce the tyrosine phosphorylation of EGFR (Kishida *et al*, 2005), which are considered to be involved in the development of drug resistance to irinotecan. Therefore, the blockage of the EGFR signalling in combination with SN38 may contribute to anti-tumour effects against irinotecan-refractory gastric cancer.

In the present study, we evaluated whether the combination of SN38 with either gefitinib or lapatinib might be useful for the treatment of chemoresistant gastric cancer cells, and if so, via what mechanism. Our results showed that both these EGFR inhibitors reversed the resistance to irinotecan and enhanced the anti-tumour efficacy of SN38.

## Materials and methods

### Chemicals

A stock solution of small-synthetic molecules, gefitinib (a TKI against EGFR; Sigma, St Louis, MO, USA) and lapatinib (GW572016; a TKI against EGFR and HER2; Toronto Research Chemicals, ON, Canada), at 37 mM in dimethyl sulfoxide was stored at −20 °C, and diluted to the desired concentration by Dulbecco's modified Eagle's medium (Nikken Biomedical Laboratory, Kyoto, Japan) before the experiment was done.

### Chemotherapeutic agents

Five chemotherapeutic drugs, irinotecan (SN38; Yakult, Tokyo, Japan), oxaliplatin (OXA; Yakult), 5-fluorouracil (5-FU; Kyowa Hakko, Tokyo, Japan), paclitaxel (PTX; Bristol-Myers, Wallingford, CT, USA), and gemcitabine (GEM; Eli Lilly, Kobe, Japan), were used in this study. All were used according to the protocol provided by the manufacture. SN38 (Yakult) was dissolved by 1 mM natrium hydroxydatum at the concentration of 1 M, stored at −20 °C, and diluted to the desired concentration by medium at the pH from 7.0 to 7.4.

### Cell lines

OCUM-2M (Yashiro *et al*, 1995) and OCUM-8 (Takemura *et al*, 2004) were derived from diffuse-type human gastric tumours. A panel of human gastric cancer cell lines including both the parental and chemoresistant sublines was used. Ten gastric cancer cell sublines resistant to SN38, OXA, 5-FU, PTX, and GEM were, respectively, established from two parent scirrhous gastric cancer cell lines, OCUM-2M and OCUM-8, by stepwise exposure to each of the five anti-cancer drugs. The drug-resistant gastric cancer cell lines from OCUM-2M were named OCUM-2M/SN38, OCUM-2M/OXA, OCUM-2M/5-FU, OCUM-2M/PTX, and OCUM-2M/GEM, respectively (Zhang *et al*, 2010). In addition, drug-resistant gastric cancer cell lines were established from OCUM-8 and designated OCUM-8/SN38, OCUM-8/OXA, OCUM-8/5-FU, OCUM-8/PTX, and OCUM-8/GEM, respectively.

### Proliferation assay

The effect of chemotherapeutic drugs with or without the small-synthetic molecules (gefitinib or lapatinib) on the proliferation of gastric cancer cells was determined by 3-(4,5-dimethylthiazol-2-yl)-2, 5-diphenyltetrazoliumbromide (MTT; Sigma) assay. Cancer cells were seeded into two 96-well plates at a concentration of 5000 cells per well with culture medium exposed to each chemotherapeutic drug at different concentrations and/or gefitinib (2.5 *μ*M) or lapatinib (200 nM). After incubation for 72 h, MTT was added into each well. The formazan product of MTT was measured as absorbance at 570 nm using a microtiter plate reader (PM2004, Wako, Osaka, Japan). The percentage of cell viability was determined as the ratio of the absorbance of the sample *vs* the control. Three independent experiments were performed. The IC_50_ of chemotherapeutic drug was determined as each chemotherapeutic drug concentration showing 50% cell growth inhibition as compared with the control cell growth. Six replicate wells were used for each drug concentration and the testing was carried out independently three times. The potential synergy between the small-molecule kinase inhibitors and 5-FU was evaluated, using the multiple drug-effect analysis with CalcuSyn software (Version 2.0, Biosoft, Cambridge, UK) including the combination index (CI) method of Chou and Talalay (1984), in which the log10 CI indicates synergism: (log CI<0), additive effect: (log CI=0) or antagonism: (log CI>0).

### Apoptosis assay

Apoptosis in response to SN38 in the presence or absence of gefitinib was examined using flow cytometry by staining the cells with annexin V-FITC and propidium iodide (Medical and Biological Laboratories, Nagoya, Japan) labelling. Cells were inoculated in 100-mm dishes at a concentration of 1.0 × 10^5^ cells ml^–1^ with SN38 (at concentration of IC_50_) and/or the gefitinib (2.5 *μ*M). After incubation for 72 h, cells were harvested and stained with annexin V-FITC and propidium iodide, and analysed by FACScan flow cytometry (Becton Dickinson, Mountain View, CA, USA). Three independent experiments were performed.

### Cell-cycle test

The cell-cycle phase distribution was evaluated using flow cytometry. Cancer cells (2 × 10^4^ cells) were seeded into a 100-mm dish with vehicle or EGFR TKI for cell-cycle test. After incubation for 72 h, the cells were harvested and managed according to the instructions of the Cycle TEST PLUS DNA reagent kit protocol (Becton Dickinson), then incubated with ribonuclease A for 10 min at room temperature, and with propidium iodide for 30 min in the dark on ice. The sub-G0/G1, S, and G2/M-phase fractions of 2 × 10^4^ cells were determined by flow cytometry using a FACScaliber (Becton Dickinson). The results were analysed using the Modofit software program (Becton Dickinson).

### Reverse transcription–PCR

The total cellular RNA was extracted using Trizol reagent (Invitrogen, Carlsbad, CA, USA) according to the manufacturer's protocol. After the genomic DNA was removed by DNAse, cDNA was prepared from 2 *μ*g of RNA with Maloney mouse leukaemia virus reverse transcriptase (Invitrogen) using random primers (Invitrogen). To investigate the influence of SN38 on the expression of *EGFR* and *HER2* in gastric cells, exponential growing cells without or with SN38 at IC_50_, respectively, were seeded into 100-mm dishes at a concentration of 3.0 × 10^5^ cells ml^–1^, and incubated for further 24 h before cell harvest. For the examination of expression at the mRNA level of apoptosis-related genes, including *Capasase-6*, *p*53, death-associated protein kinase-1 (*DAPK-1*), *DAPK-2*, and *DAPK-3*, and SN38 metabolism-related genes including *UGT1A1* and *ABCG2*, cancer cells were incubated for 24 h in the presence or absence of lapatinib or gefitinib. Quantitative real-time RT–PCR was done on the ABI Prism 7000 (Applied Biosystems, Foster City, CA, USA) using the commercially available gene expression assay for *EGFR* (Hs01076091), *HER2* (Hs01001580), *Capasase-6* (Hs00154250), *p*53 (Hs01034249), *DAPK-1* (Hs00234489), *DAPK-2* (Hs00204888), *DAPK-3* (Hs00154676), *UGT1A1* (Hs01053796), and *ABCG2* (Hs02511055). PCR was performed at 95 °C for 15 s and 60 °C for 60 s for 40 cycles. As internal standard to normalise mRNA levels for differences in sample concentration and loading, amplification of *glyceraldehyde-3-phosphate dehydrogenase* was used. The threshold cycle (*C*_t_) values were used for calculation of the relative expression ratios between control and treated cells using the formula described by Pfaffl (2001). All quantitative PCR reactions were done in triplicate.

### Western blot analysis

Cell lysates were collected after different treatments. After the protein concentration of each sample was adjusted, electrophoresis was carried out using 10% Tris/Gly gels (Invitrogen Life Technologies, Inc., Gaithersburg, MD, USA). The protein bands obtained were transferred to an Immobilon-P Transfer membrane (Amersham, Aylesbury, UK). The membrane was kept in PBS-T (10 mM PBS and 0.05% Tween 20) supplemented with 5% bovine albumin (Sigma) at room temperature for 1 h. Then, the membrane was placed in PBS-T solution containing each primary antibody: EGFR (Millipore, Billerica, MA, USA), ErbB2 (Dako, Cambridge, UK), cleaved caspase-6 (Assay Biotechnology, Sunnyvale, CA, USA), and p53 (Dako), and DAPK-2, and allowed to react at room temperature for 2 h. The levels of specific proteins in each lysate were detected by enhanced chemiluminescence using ECL plus (Amersham) followed by autoradiography.

### Animal models

BALB/c nude mice (Clea Japan, Shizuoka, Japan) were used. All experiments with nude mice were performed in accordance with the animal experiments guidelines approved by Osaka City University Ethical Committee. Xenografts were established by injecting 1 × 10^7^ OCUM-2M/SN38 cells into the flanks of mice at 4 weeks of age. Mean tumour size was observed to be 60 mm^2^ at 10 days after inoculation. Accordingly, 10 mg kg^–1^ per day of SN38, and/or 30 mg kg^–1^ per day of Lapatinib was administered for 5 days per week for 3 weeks, except in control. SN38 was intraperitoneally injected, and Lapatinib was administered orally. Tumour size (*S*) was determined at each time point by measuring length (*l*) and width (*w*), then calculating the volume (*V*=*lw*). Medication-defined groups were Vehicle (control group; *n*=8), Lapatinib (30 mg kg^–1^ per day; *n*=8), SN38 (10 mg kg^–1^ per day; *n*=8), Lapatinib combined with SN38 (*n*=8). The synergistic effect was determined when the value by the combination of lapatinib with SN38 was less than the expected value, as previously reported (Marth *et al*, 1986): The expected value (mm^2^) of the combined effects=the effects of SN38 (mm^2^) × the effects of lapatinib (mm^2^)/vehicle control (mm^2^).

### Statistical analysis

The quantitative ratios of different groups were compared using Student's *t*-test. Probability values of *P*<0.05 were regarded as statistically significant. All statistical tests were two sided.

## Results

### Combination effects of an EGFR inhibitor and anti-cancer drugs on the proliferation of gastric cancer cells

**Figure 1:**
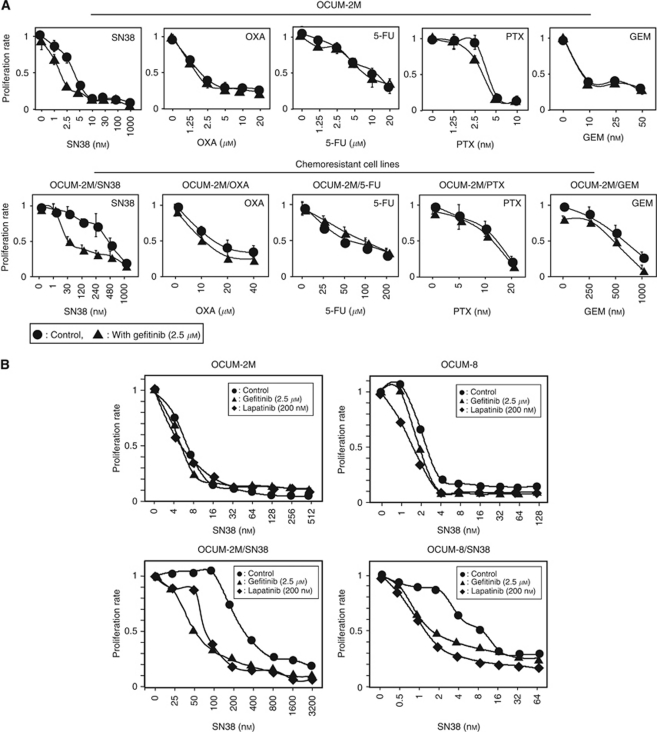
Growth-inhibitory effects of gefitinib or lapatinib with SN38 in gastric cancer cell lines. (**A**) A human gastric cancer cell line, OCUM-2M, and chemoresistant sublines were exposed to each chemotherapeutic drug at different concentrations in the absence (•) or presence of gefitinib (▴). Gefitinib increased the anti-proliferation effect of SN38 in SN38-resistant cell line, OCUM-2M/SN38, but not other cell lines. (**B**) Gefitinib and lapatinib decreased the IC_50_ values of SN38-resistant cell lines, OCUM-2M/SN38 and OCUM-8/SN3, but not that of parent cell lines, OCUM-2M and OCUM-8. Gastric cancer cells were exposed to SN38 at various concentrations in the absence (•) or presence of gefitinib (▴) or lapatinib (♦).

Figure 1A shows the anti-proliferative effect of each of the five anti-cancer drugs, SN38, OXA, 5-FU, PTX, or GEM, in combination with gefitinib in OCUM-2M cells and the respective daughter cell lines resistant to each of the five chemotherapeutic drugs. Gefitinib increased the anti-proliferation effect of SN38 in OCUM-2M/SN38 cells, but not in the other cell lines. With respect to the effects on OCUM-8/SN38 and the respective daughter chemoresistant cell lines, gefitinib increased the anti-proliferation effect of SN38 in OCUM-8/SN38 cells, but not in the other cell lines (data not shown).

Figure 1B shows the cell growth inhibition curve of SN38 in the presence or absence of 2.5 *μ*M gefitinib or 200 nM lapatinib in OCUM-2M, OCUM-2M/SN38, OCUM-8, and OCUM-8/SN38 cells. The IC_50_ value (the drug concentration needed for 50% growth reduction on the survival curve) of SN38-resistant cell lines and their parent cell lines to SN38 was summarised in Table 1. The IC_50_ value for SN38-resistant sublines, OCUM-2M/SN38 (304 nM) and OCUM-8/SN38 (10.5 nM), was higher than that of parent cell lines, OCUM-2M (6.4 nM) and OCUM-8 (2.6 nM). The resistance index (RI) was calculated as the ratio of the IC_50_ of the drug-resistant cell line to the IC_50_ of parent cell line. The RI values of OCUM-2M/SN38 and OCUM-8/SN38 cells against SN38 were 47.5 and 4.0, respectively. The RI values of OCUM-2M/SN38 and OCUM-8/SN38 cells against SN38 were both >3.0, confirming that each subline was resistant to SN38.

The IC_50_ value for OCUM-2M/SN38 was decreased by co-exposure to SN38 and gefitinib (50 nM), and co-exposure to SN38 and lapatinib (78 nM), in comparison with SN38 alone (304 nM). Taken together, the IC_50_ values for OCUM-8/SN38 cells was decreased by co-exposure to SN38 and gefitinib (1.6 nM), and co-exposure to SN38 and lapatinib (1.3 nM), in comparison with SN38 alone (10.5 nM). On the other hand, neither EGFR inhibitor (gefitinib at 2.5 *μ*M or lapatinib at 200 nM) significantly suppressed the proliferation of any of the cell lines in this study when used alone.

### Synergistic effects of EGFR inhibitors on the anti-proliferative efficiency of SN38

**Figure 2:**
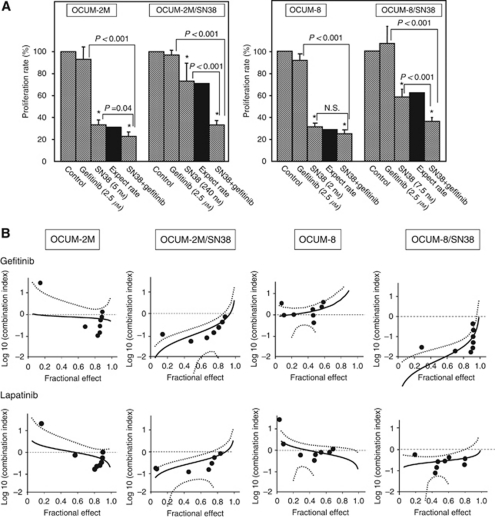
Synergistic effect of gefitinib or lapatinib with SN38 in gastric cancer cell lines. (**A**) The combination with gefitinib, and SN38 significantly suppressed cancer cell proliferation in OCUM-2M, OCUM-2M/SN38, and OCUM-8/SN3 cells in comparison to the SN38 alone, but not OCUM-8 cells. The expected value of the combined effects (%) was defined as: effects of SN38 alone/control × effects of gefitinib alone/control × 100%. The proliferation of SN38-resistant cells exposed to the combination of gefitinib plus SN38 was lower than expected. The results are presented as the mean of three independent experiments, and the bars indicate the s.d. ^*^P<0.05 *vs* control. (**B**) The CI plots derived from CalcuSyn software. The combination of gefitinib or lapatinib plus SN38 shows a synergistic (log CI <0) growth-inhibitory effect for OCUM-2M/SN38 and OCUM-8/SN38 cells, but antagonistic effect (log CI >0) was found at some points in OCUM-2M and OCUM-8 cells. dotted line, log (CI)=0.

Figure 2A shows the effects of the EGFR inhibitors on the anti-proliferative efficiency of SN38. In OCUM-2M cells, the proliferation rates of gefitinib, SN38 (5 nM), and gefitinib with SN38 were 93%, 32.6%, and 24.8%, respectively. In OCUM-2M/SN38 cells, the cell growth rates after exposure to gefitinib, SN38 (240 nM), or gefitinib plus SN38 were, respectively, 97%, 74%, and 34%, from which it could be concluded that gefitinib clearly inhibited the cell growth of OCUM-2M/SN38 when administered in combination with SN38. In OCUM-8 cells, the proliferation rates after administration of gefitinib, SN38 (5 nM), or gefitinib with SN38 were, respectively, 93%, 32%, and 29%. In OCUM-8/SN38 cells, the growth rates following administration of gefitinib, SN38 (240 nM), and gefitinib plus SN38 were, respectively, 108%, 59%, and 36%, from which it could be concluded that the combination treatment clearly inhibited the growth of OCUM-2M/SN38 cells. The expected value of the combined effects (%) was defined as: effects of SN38 alone/control × effects of gefitinib alone/control × 100%. The combination effects of gefitinib with SN38 were synergistic, in both the SN38-resistant cell lines and OCUM-2M because the proliferation rate associated with the combination of SN38 and gefitinib (24.8%, 34%, 29%, and 36%) was less than the expected value (30.3%, 71.8%, 30%, and 63.7%) in OCUM-2M, OCUM-2M/SN38, OCUM-12, and OCUM-12/SN38 cells, respectively. The proliferation of SN38-resistant cells exposed to the combination of gefitinib plus SN38 was lower than expected (Figure 2A). Figure 2B shows the plotted combination results of SN38 with gefitinib or lapatinib at various concentrations. When data were analysed by the CalcuSyn software program, the effects of these combinations of EGFR inhibitors with SN38 were synergistic (log CI <0) in both the SN38-resistant cell lines OCUM-2M/SN38 and OCUM-8/SN38. In contrast, antagonistic effect (log CI>0) was found at >128 nM SN38 and 16 nM SN38 with the combination in the parent cell lines, OCUM-2M and OCUM-8, respectively.

### Effects of SN38 on EGFR or HER2 gene expression in gastric cancer cells

**Figure 3:**
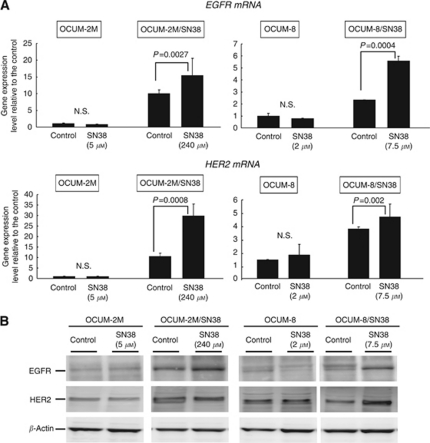
Effects of SN38 on the expression of *HER* family by reverse transcription–PCR. (**A**) SN38 increased the expression of *EGFR* and *HER2* in OCUM-2M/SN38 and OCUM-8/SN38, but not in OCUM-2M and OCUM-8. (**B**) The expression of EGFR and HER2 was increased by SN38 in OCUM-2M/SN38 and OCUM-8/SN38 cells.

In OCUM-2M/SN38 and OCUM-8/SN38 cells, the expression levels of *EGFR* and *HER2* were higher than those in the parent OCUM-2M and OCUM-8 cells. The expression levels of *EGFR* and *HER2* was significantly increased by SN38 in OCUM-2M/SN38 and OCUM-8/SN38 cells, but not in OCUM-2M and OCUM-8 cells (Figure 3A). Taken together, the protein levels of EGFR and HER2 were increased by SN38 in OCUM-2M/SN38 and OCUM-8/SN38 cells (Figure 3B).

### Effect of SN38 on the apoptosis of cancer cells in the presence or absence of EGFR inhibitors

**Figure 4:**
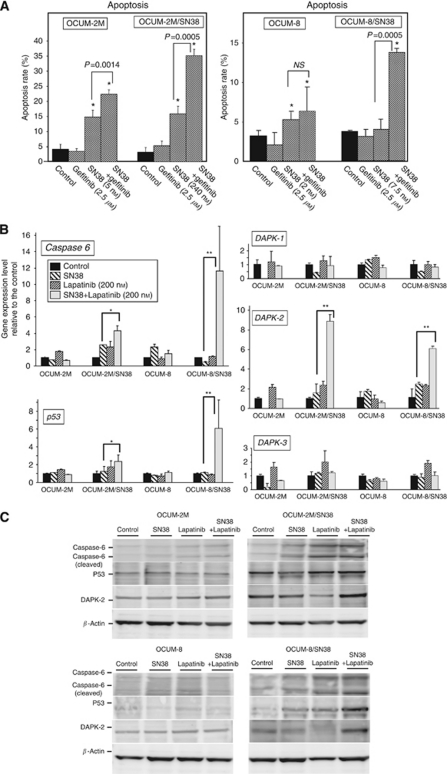
Apoptosis induction by gefitinib and/or SN38 in gastric cancer cells. (**A**) SN38 was added at the concentration of IC_50_. In all of the four cell lines tested, no significant difference of the apoptosis rate was found between gefitinib (2.5 *μ*M) treatment and control. In contrast, the apoptosis rate induced by the combination of gefitinib and SN38 was significantly increased in comparison to the SN38 alone in the OCUM-2M, OCUM-2M/SN38, and OCUM-8/SN38 cells. ^*^P<0.05 *vs* control. (**B**) Lapatinib with SN38 significantly increased the mRNA expression levels of *caspase-6*, *p53*, and *DAPK-2* compared with exposure to SN38 alone or lapatinib alone in SN38-resistant cells, OCUM-2M/SN38 and OCUM-8/SN38, but not in OCUM-2M and OCUM-8 cells. ^*^P<0.05 *vs* control; ^**^P<0.01 *vs* control. (**C**) SN38 plus lapatinib increased caspase-6, p53, and DAPK-2 expression, compared with either alone, in SN38-resistant cells.

Figure 4A shows the rates of apoptosis induced by SN38, gefitinib (2.5 *μ*M), or both. The apoptosis rates of the control group and the gefitinib (2.5 *μ*M) monotherapy group were 4.2% and 3.5% in OCUM-2M cells, and 3.2% and 5.3% in OCUM-2M/SN38 cells, respectively. The apoptosis rates of the control group and gefitinib monotherapy group were 3.3% and 2.1% in OCUM-8 cells, and 3.8% and 3.2% in OCUM-8/SN38 cells, respectively. No significant difference in apoptosis rates was observed between the control group and gefitinib group in any of the gastric cancer cell lines. When OCUM-2M was co-exposed to SN38 (5 nM) and gefitinib (2.5 *μ*M), the apoptosis rate was significantly (*P*<0.01) increased to 22.4%, compared with that (14.8%) after exposure to SN38 alone. In OCUM-2M/SN38 cells, the apoptosis rates achieved by the combination of SN38 (240 nM) and gefitinib was significantly (*P*<0.001) increased to 35.1%, compared with the apoptosis rate (15.9%) by SN38 (240 nM) alone. In OCUM-8 cells, the combination of SN38 (2 nM) and gefitinib (2.5 *μ*M) induced apoptosis at a rate of 6.4% in comparison to the rate of 5.3% induced by SN38 (2 nM) alone. In OCUM-8/SN38 cells, gefitinib plus SN38 (7.5 nM) achieved a 13.8% rate of apoptosis, which was significantly higher than the rate of 4.1% induced by SN38 (7.5 nM) alone (*P*<0.001). In contrast, there was no significant difference in the rates of apoptosis by SN38 (at IC_50_) monotherapy between the parent cell lines and SN38-resistant cell lines. Taken together, lapatinib also significantly increased apoptosis rates in SN38-resistant cell lines (data not shown).

### Effects of SN38 plus an EGFR inhibitor on the expression of apoptosis-related molecules

In OCUM-2M/SN38 and OCUM-8/SN38 cells, the mRNA expression levels of *caspase-6*, *p53*, and *DAPK-2* were significantly increased when cells were cultured in the presence of SN38 plus lapatinib (200 nM), compared with exposure to SN38 alone or lapatinib alone. In contrast, there were no significant differences in the expression levels of these genes between the SN38 monotherapy and the SN38 plus lapatinib treatment in either OCUM-2M or OCUM-8 cells (Figure 4B). The protein levels of caspase-6, p53, and DAPK-2 were increased in the presence of SN38 plus lapatinib, compared with exposure to SN38 alone or lapatinib alone, in OCUM-2M/SN38 and OCUM-8/SN38 cells (Figure 4C).

### Effects of EGFR inhibitors on the cell cycle

**Figure 5:**
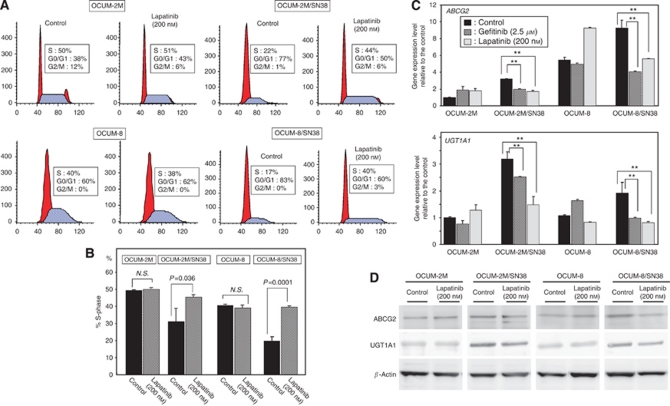
Effects of EGFR inhibitors on the cell cycle and SN38 metabolism-related genes. (**A**) The representative FACS-based cell-cycle analyses. (**B**) The percentage of cells in the S-phase in OCUM-2M/SN38 and OCUM-8/SN38 cells was significantly increased following the addiction of lapatinib (200 nM), compared with the control. N.S.=not significant. (**C**) EGFR inhibitor, lapatinib and gefitinib, significantly increased the mRNA expression levels of *UGT1A1* and *ABCG2* in OCUM-2M/SN38 and OCUM-8/SN38 cells. In contrast, in OCUM-2M and OCUM-8 cells, the effect by EGFR inhibitors on the expression levels of *UGT1A1* and *ABCG2* were not maintained the same. ^**^P<0.01 *vs* control. (**D**) ABCG2 and UGT1A1 expression in SN38-resistant cells were higher than those in the parent cells, and was decreased by lapatinib in SN38-resistant cells (**D**).

The representative FACS analyses data were shown in Figure 5A. The percentage of cells in the S-phase was low in OCUM-2M/SN38 and OCUM-8/SN38 cells, in comparison with that in OCUM-2M and OCUM-8 cells. The EGFR inhibitor lapatinib increased the percentage of cells in the S-phase in OCUM-2M/SN38 and CUM-8/SN38 cells, but not in OCUM-2M and OCUM-8 cells. Lapatinib decreased the percentage of cells in the G0/G1 in OCUM-2M/SN38 and CUM-8/SN38 cells (Figure 5B).

### Effects of EGFR inhibitors on SN38 metabolism-related genes, UGT1A1 and ABCG2

The expression levels of *ABCG2* and *UGT1A1*in OCUM-2M/SN38 and OCUM-8/SN38 cells were higher than those in the parent OCUM-2M and OCUM-8 cells. In OCUM-2M/SN38 and OCUM-8/SN38 cells, the EGFR inhibitors gefitinib and lapatinib significantly decreased the mRNA expression levels of *ABCG2* and *UGT1A1*, in comparison with the vehicle control. In contrast, lapatinib increased the expression levels of *ABCG2* in OCUM-2M cells and OCUM-8 cells. Gefitinib increased the expression levels of *UGT1A1* in OCUM-8 cells (Figure 5C). The protein levels of ABCG2 and UGT1A1in OCUM-2M/SN38 and OCUM-8/SN38 cells were higher than those in the parent OCUM-2M and OCUM-8 cells, and was decreased by lapatinib in OCUM-2M/SN38 and OCUM-8/SN38 cells (Figure 5D).

### Effect of Lapatinib and/or SN38 on tumour development *in vivo*

**Figure 6:**
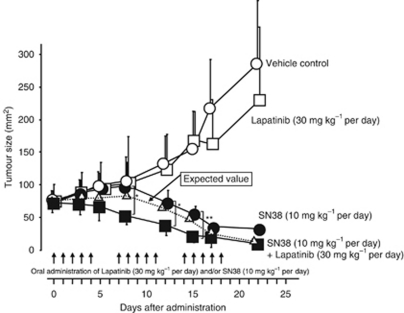
Effect of the combination of SN38 with Lapatinib on the proliferation of xenografted tumour *in vivo*. The tumour by OCUM-2M/SN38 cells was significantly decreased by SN38 plus Lapatinib (▪) compared with the size resulting from SN38 alone treatment (•). The significant difference of tumour size between SN38 alone and SN38 with the Lapatinib was found between day 8 and day 15 after administration. The tumour size by the combination of Lapatinib and SN38 was lower than the expected value (Δ) between day 8 and day 15 after administration. In contrast, no significant difference of tumour size between vehicle control (○) and Lapatinib alone treatment (□) was found. ^*^P<0.05; ^**^P<0.01. The expected value of the combined effects=the effects of SN38 × the effects of lapatinib/vehicle control (dotted line).

The mean volumes of the subcutaneous tumour of the control, Lapatinib, SN38, Lapatinib, plus SN38 groups were 101, 98, 96, and 60 mm^2^ at day 8 after administration, respectively. The size of the tumours in mice receiving the combination of SN38 with the Lapatinib was significantly (*P*<0.01) smaller than in those receiving either SN38 or Lapatinib alone. The significant difference of tumour size between SN38 alone and SN38 with the Lapatinib was found between day 8 and day 15 after administration. The tumour size by the combination of lapatinib and SN38 was lower than the expected value, evaluating that the combination of Lapatinib and SN38 shows a synergistic effect. In contrast, no significant difference of tumour size between control and Lapatinib was found (Figure 6).

## Discussion

The combination of an EGFR inhibitor, lapatinib or gefitinib, with SN38 achieved a greater suppression of cancer cell proliferation in all four cell lines tested than monotherapy with either agent alone. Previous studies have shown that lapatinib may be effective in combination with irinotecan as a chemotherapeutic treatment for chemonative gastrointestinal cancer cells (LaBonte *et al*, 2009). This is supported by our observation that the proliferation of parent gastric cancer cells was more highly decreased by the combination of an EGFR inhibitor and SN38 than by treatment with SN38 alone. Moreover, the combination of an EGFR inhibitor and SN38 synergistically suppressed cell growth and induced apoptosis in the SN38-resistant gastric cancer cell lines, OCUM-2M/SN38 and OCUM-8/SN38, but not in OCUM-2M and OCUM-8 cells. Taken together, the *in vivo* tumour by OCUM-2M/SN38 cells was significantly decreased by SN38 plus Lapatinib compared with the size resulting from SN38 alone. These findings suggested that EGFR inhibitors might be therapeutically promising for irinotecan-refractory gastric carcinomas when used in combination with SN38. Clinical studies have shown that the combination of irinotecan and the EGFR inhibitor cetuximab is preferable for patients with irinotecan-refractory colorectal cancer (Cunningham *et al*, 2004; Saltz *et al*, 2004; Vincenzi *et al*, 2006). In gastric cancer, EGFR has become the target of choice (Kim *et al*, 2008), while EGFR inhibitors have proven only weakly effective (Namiki *et al*, 2006; Becker *et al*, 2006b). Further clinical studies will be needed to determine whether EGFR inhibitors reverse the resistance to irinotecan in patients with advanced gastric cancer.

The present study indicated that SN38 upregulated the expression levels of *EGFR* and *HER2* in SN38-resistant gastric cancer cells, but not parent cells. Increased EGFR/HER2 expression may be a survival response by some tumours exposed to chemotherapeutic agents. The activation of EGFR could have an important role for the cell proliferation and the inhibition of apoptosis (Kishida *et al*, 2005), indicating that the SN38-induced activation of the EGF/EGFR may be involved in the resistance of gastric cancer cells to irinotecan. SN38-resistant cancer cells might be more dependent on EGFR signalling pathways for survival, and therefore may become more sensitive to EGFR inhibitors. The different effects of SN38 treatment on EGFR expression might explain the different effects of the EGFR inhibitor plus SN38 combination treatment between the parent cells and resistant cells. Several studies have reported that tumour cell lines with acquired resistance to chemotherapeutic drugs can develop increased expression of EGFR (Dai *et al*, 2005; Kishida *et al*, 2005; Wang *et al*, 2007). Kishida *et al* (2005) suggested that the mechanisms responsible for the SN38-induced activation of the EGFR might include the generation of reactive oxygen species and the activation of protein kinase C, followed by metalloproteinase activation and the sequential ectodomain shedding of EGFR ligands. In addition, Wang *et al* (2007) suggested that the chemotherapy-induced EGFR activation is regulated by heparin-binding EGF-like growth factor (HB-EGF). These mechanisms might be associated with the upregulation of EGFR and HER2 in the SN38-resistant gastric cancer cell lines.

Gefitinib at a concentration of 2.5 *μ*M and lapatinib at 200 nM were used in each assay. In our previous study, different concentrations of gefitinib ranging from 0.1 to 4 *μ*M, or lapatinib ranging from 50 to 400 nM have been applied, and the slight growth inhibiting ability of gefitinib was found at each concentration. Since 2.5 *μ*M gefitinib and 200 nM lapatinib showed no significant effect on the proliferation of gastric cancer cell lines, we used the concentration at each assay. Our study indicated that either EGFR inhibitor at low concentration suppressed the proliferation of cancer cells in combination with SN38. In contrast, the antagonistic effect (log CI>0) was found at >128 nM SN38 and 16 nM SN38 with the combination in the parent cell lines, OCUM-2M and OCUM-8, respectively, but not found in the resistant cell lines. Because the IC_50_ value of SN38 for OCUM-2M and OCUM-8 was 6.4 and 2.6 nM, respectively, the antagonism was shown only at high concentration of SN38 (more than five times of IC_50_). These findings might suggest that the combination of gefitinib or lapatinib might reduce the anti-tumour effects of SN38 at very high concentration of SN38, while these concentrations were not clinical practice. Although the toxicity of irinotecan or an EGFR inhibitor is predictable, dose adjustment should be made when necessary. The combination of EGFR inhibitor and SN38 at a low dose might reverse SN38 resistance, and could be useful for patients experiencing toxicity from SN38.

The combination of SN38 and an EGFR inhibitor significantly increased apoptosis rates in SN38-resistant cell lines, compared with SN38 alone. These findings suggested that the synergistic anti-proliferative effect of the EGFR inhibitors used in this study might be explained by an enhancement of apoptosis. Irinotecan causes S-phase-specific cell killing by poisoning topoisomerase I (Topo I) in the cells. In the present study, the percentage of cells in the S-phase was low in the SN38-resistant cell lines, and the EGFR inhibitors increased the percentage of cells in the S-phase in the SN38-resistant cell lines, but not in the parent cells. These findings suggested that upregulation of the S-phase in the SN38-resistant cell lines by the EGFR inhibitors might be one of the mechanisms responsible for the increase of apoptosis in the combination therapy with SN38.

Several studies have been done to uncover possible mechanisms for the cellular resistance to irinotecan. Dai *et al* (2008) reported that lapatinib reverses ABCB1- and ABCG2-mediated tumour multidrug resistance by directly inhibiting their transport function. Our study also showed that EGFR inhibitors significantly decreased ABCG2 levels in SN38-resistant cells, but not parent cells. Moreover, the expression level of UGT1A1, which catalyses the glucuronidation of SN38, was decreased by the combination treatment. The downregulation of SN38 metabolism-related genes, ABCG2 and UGT1A1, by EGFR inhibitors may also be one of the mechanisms involved in the synergistic effects between the EGFR inhibitors and irinotecan in SN38-resistant cells.

The combination of an EGFR inhibitor and SN38 upregulated the expression level of apoptosis-related molecules, caspase-6, p53, and DAPK-2, in both SN38-resistant cell lines. The caspase family comprises enzymes engaged in cell apoptosis (Korzeniewska-Dyl, 2008). Caspase-6 signalling induced by an EGFR inhibitor might contribute to the induction of apoptosis in SN38-resistant cancer cells. *p53*, a nuclear phosphoprotein, has an important role in apoptosis, growth arrest, genomic stability, cell senescence, and differentiation. In A549 cells, gefitinib induces apoptosis through a *p53*-dependent upregulation of pro-apoptotic molecules and downregulation of anti-apoptotic molecules (Chang *et al*, 2008). In some non-small cell lung cancers without *EGFR* mutations, *p53* had a role in determining gefitinib sensitivity (Rho *et al*, 2007). In our study, *p53* expression was only clearly increased in SN38-resistant cells. Epidermal growth factor receptor inhibitors regulated p53 signalling in SN38-resistant cell lines. The DAPK family is associated with apoptotic or autophagic cell death signals in response to various cellular stresses (Kogel *et al*, 2001; Gozuacik and Kimchi, 2006), and its members can induce apoptosis in a *p53*-dependent manner (Kogel *et al*, 2003). In our study, elevated expression of *DAPK-2* was observed in both OCUM-2M/SN38 and OCUM-8/SN38 cells, while there were no changes in DAPK-1 or DAPK-3 expression. The upregulation of caspase-6, p53, and DAPK-2 might take part in the synergistic effect between the EGFR inhibitors and SN38, and might result in the synergistic induction of apoptosis of irinotecan-resistant gastric cancer cells.

Epidermal growth factor receptor inhibitors achieved a better clinical response rate for colorectal carcinomas when used in combination with cytostatic drugs, including irinotecan, 5-FU, and OXA (Yen *et al*, 2010; Folprecht *et al*, 2010). Our study found that the combination of an EGFR inhibitor with either 5-FU or OXA did not exhibit a synergistic effect, but the combination of an EGFR inhibitor with irinotecan did. The combination of an EGFR inhibitor with irinotecan might be more important for anti-cancer therapy than that with 5-FU or OXA. Caspase-6, p53, DAPK-2, ABCG2, and UGT1A1 might be useful surrogate markers to predict better clinical response by an EGFR inhibitor in combination with irinotecan.

In conclusion, EGFR inhibitors increased the sensitivity of SN38 in gastric cancer cells, especially in SN38-resistant gastric cancer cells. The mechanism of this synergism by EGFR inhibitors might be responsible for the upregulation of caspase-6, p53, and DAPK-2 and the downregulation of ABCG2 and UGT1A1 in SN38-resistant gastric cancer cells, resulting in the induction of apoptosis of irinotecan-resistant cells. The combination treatment with an EGFR inhibitor and irinotecan might be a therapeutically promising approach for irinotecan-refractory gastric carcinomas.

## Figures and Tables

**Table 1:** IC_50_ values of SN38-resistant cell lines and their parent cell lines to SN38

	**IC_50_ values for SN38**		
**Cell line**	**SN38 alone**	**SN38 with gefitinib (2.5 *μ*M)**	**SN38 with lapatinib (200** **nM)**
OCUM-2M	6.4 nM	5.4 nM	5.4 nM
OCUM-2M/SN38	304 nM	50 nM	78 nM
OCUM-8	2.6 nM	1.9 nM	1.6 nM
OCUM-8/SN38	10.5 nM	1.6 nM	1.3 nM

IC_50_ was determined as a concentration causing 50% growth inhibition for each cell line.
